# Evaluating the efficacy of intensified insulin therapy in hypertriglyceridemic acute pancreatitis management: a systematic review and meta-analysis

**DOI:** 10.3389/fmed.2026.1944464

**Published:** 2026-09-17

**Authors:** Haifu Zhang, Shuojun Li, Yonghang Feng, Biyun Xie

**Affiliations:** Department of Gastroenterology, Fuyang Campus of Zhejiang Provincial People's Hospital (The First People's Hospital of Fuyang), Hangzhou, Zhejiang, China

**Keywords:** blood purification, hypertriglyceridemic acute pancreatitis, intensive insulin therapy, lipid management, triglyceride

## Abstract

**Background:**

Hypertriglyceridemia has long been regarded as a major cause of pancreatitis, and its incidence has been increasing annually. However, the role of intensified insulin therapy in treating hypertriglyceridemic acute pancreatitis (HTG-AP) remains debatable.

**Methods:**

A prospectively registered meta-analysis (CRD420261344642) was conducted to examine eligible randomized controlled trials (RCTs) or retrospective observational studies (ROSs) through searches across PubMed, Cochrane Library, and Embase databases, as well as the Web of Science, with a search period up to 1st March 2026. Eligible meta-analyses were selected based on predefined criteria. Data analysis was conducted using RevMan (version 5.4) and R (version 4.4.3).

**Results:**

A total of 12 studies including 1,505 patients were identified that compared the effectiveness of intensive insulin therapy with other treatment modalities in patients with HTG-AP. The results revealed a statistically non-significant difference in triglyceride reduction between intensive insulin therapy and other treatments (intensive insulin therapy versus other therapy: 24hTG, MD: 1.25, 95%CI: −0.53 to 3.03, *P* = 0.17; 48hTG, MD: 0.07, 95%CI: −0.79 to 0.93, *P* = 0.88; 72hTG, MD: −0.54, 95%CI: −3.36 to 2.27, *P* = 0.71. Similarly, triglycerides were not significantly reduced with blood purification therapy (intensive insulin therapy versus blood purification: 24hTG, MD: 1.47, 95%CI: −1.24 to 4.19, *P* = 0.29; 48hTG, MD: 0.72, 95%CI: −0.69 to 2.13, *P* = 0.32; 72hTG, MD: 1.89, 95%CI: −2.22 to 6.00, *P* = 0.37). Additionally, intensive insulin therapy was not associated with a higher incidence of local pancreatic complications or systemic adverse effects. Intensive insulin therapy experienced a significantly reduced duration of hospital stay in comparison to the cohort receiving the blood purification regimen (MD: −3.60, 95%CI: −5.09 to −2.11, *P* < 0.01).

**Conclusions:**

Current evidence does not demonstrate the superiority of either intensive insulin therapy or blood purification, but intensive insulin therapy may shorten the length of hospital stay to a certain extent.

**Systematic Review Registration:**

https://www.crd.york.ac.uk/PROSPERO/view/CRD420261344642, PROSPERO CRD420261344642.

## 1 Introduction

Acute pancreatitis (AP) is a common clinical emergency affecting multiple organs and can be caused by factors such as gallstones, alcohol use, and high triglyceride levels (1). The global incidence of AP ranges from 3.4 to 73.4 cases per 100,000 individuals and is increasing annually by 2%–5% (2). Historically, hypertriglyceridemia has been considered the third leading cause of pancreatitis, following alcohol consumption and gallstones, and accounts for approximately 10% of all cases (3). Nevertheless, the incidence of this condition continues to increase annually among Asian populations due to lifestyle changes. In China, it has emerged as the second leading cause, accounting for over 25% of AP cases (4, 5).

AP resulting from hypertriglyceridemia is characterized by more rapid progression and a higher incidence of systemic inflammatory response syndrome, persistent organ dysfunction, local complications, and mortality (6). Consequently, early diagnosis and intervention are crucial; however, a standardized treatment protocol for hypertriglyceridemic acute pancreatitis (HTG-AP) remains lacking. HTG-AP management primarily involves fluid resuscitation, organ support, and reduction of blood triglyceride levels through pharmacological and non-pharmacological interventions (7).

Non-pharmacological interventions, such as hemofiltration, hemoperfusion, and plasma exchange, have been reported to rapidly decrease triglyceride (TG) levels and inflammatory markers. However, the comparative efficacy of these interventions relative to conventional therapies remains a subject of debate (8). Pharmacological approaches include oral lipid-lowering agents and the administration of low-molecular-weight heparin and insulin (9). Insulin may improve the overall prognosis for patients with HTG-AP by ameliorating glucose metabolism disorders and mitigating the production of free radicals associated with these metabolic disturbances (10). Insulin therapy involves administering insulin via subcutaneous injections at set doses and through intensified insulin therapy. Intensified insulin therapy (IIT) appears to be a safe and effective method for lowering triglyceride levels in HTG-AP patients, achieving an 85.2% reduction at discharge compared to 79.8% with subcutaneous insulin (11). A recent RCT found that adding low-molecular-weight heparin to insulin therapy for patients with HTG-AP is no more effective than intensified insulin therapy alone, suggesting heparin may be unnecessary (12). Consequently, the objective of this study was to evaluate the efficacy of intensified insulin therapy compared to alternative treatments for patients with HTG-AP.

## 2 Methods

This systematic review and meta-analysis were conducted in accordance with the Preferred Reporting Items for Systematic Reviews and Meta-Analyses (PRISMA) guidelines (13) and mostly followed a pre-specified protocol registered on PROSPERO with registration number CRD420261344642. To ensure a more comprehensive search, we incorporated the Web of Science database subsequent to the commencement of the research. Considering that the English databases encompasses research data globally, we limited the language of the literature to English.

### 2.1 Search strategy

The PubMed, Cochrane Library, and Embase databases, as well as the Web of Science, were comprehensively searched for articles published up to 1st March 2026. The following keywords were used: “insulin”, “Hypertriglyceridemic acute pancreatitis”, “hypertriglyceridemia-induced acute pancreatitis”, “Hypertriglyceridemia-Induced Pancreatitis”, “Hypertriglyceridemia-Associated Acute Pancreatitis”, and “hyperlipidemic severe acute pancreatitis”. See the Supplementary Material for specific search strategies.

### 2.2 Inclusion and exclusion criteria

The inclusion criteria were as follows: Clinical controlled studies on the treatment of patients with HTG-AP, including randomized controlled trials (RCTs) and retrospective observational studies (ROSs).

The inclusion criteria for this study are as follows:

Population: Diagnosis of HTG-AP is usually based on the revised Atlanta criteria, in which two of the following three features are required for the diagnosis of AP: hyperlipasemia (>3 times the upper limit of normal); acute abdominal pain; and/or signs of pancreatitis on computed tomography scan; along with a serum triglyceride level exceeding 11.3 mmol/L.

Intervention: Patients in the intervention group were treated with intensive insulin therapy alone. Intensified insulin therapy is defined as continuous intravenous infusion of short-acting insulin, with the dosage ranging from 0.1 to 0.3 U/kg/h, and 10% glucose to prevent hypoglycemia.

Comparators: Patients in the control group were treated with conventional therapy, low molecular weight heparin/heparin, blood purification therapy (including high-volume hemofiltration, plasma exchange, therapeutic plasma exchange), or other treatment regimens, either alone or in combination.

Outcomes: The main outcomes are the triglyceride levels at 24, 48, and 72 h after treatment, as well as the time to reach the target triglyceride level; the secondary outcomes are local complications of pancreatitis, systemic complications, duration of hospital stay, and hospitalization costs.

### 2.3 Outcomes assessed

Two independent reviewers extracted the following data from pre-specified reports: name of the first author, year of publication, number of participants, patient characteristics (BMI, number of drinkers or diabetics), baseline triglyceride and glucose levels, fasting protocol, fluid resuscitation strategy and outcome measures.

### 2.4 Quality assessment

The methodological quality of the included RCTs was evaluated using the Cochrane Risk of Bias tool. The quality of the included ROSs was evaluated using the Newcastle-Ottawa Scale (NOS). Study quality was assessed across three main dimensions: selection, comparability, and exposure/outcome. NOS quality scores ranged from 0 to 9, with NOS scores ≥ 6 indicating high study quality.

### 2.5 Statistical analysis

The median (interquartile range) data extracted from the original literature were converted to the mean ± standard deviation, $\bar{x}(w)=w\left(\frac{q1+q3}{2}\right)+(1-w)m$, take w = 1/2 for *n* ≤ 25 and w = 0 for *n* > 25; $S\approx \frac{q3-q1}{\eta (n)}$ (14, 15).

Simultaneously, glucose and triglyceride measurements from various studies were standardized to mmol/L, with 1 mmol/L glucose equivalent to 18.01 mg/dL and 1 mmol/L triglycerides equivalent to 88.57 mg/dL.

Statistical analyses were performed using Microsoft Excel 2016 and RevMan (version 5.4). Indirect results were compared using the Bucher method in R (version 4.4.3). Using the random effects model, dichotomous variables are expressed as risk ratios (RRs) and 95% confidence intervals (CIs). For continuous variables, the mean difference (MD) or standardized mean difference (SMD) with 95% CI was applied. The Chi-squared (X^2^) test was employed to assess heterogeneity, while the I^2^ statistic was used to quantify it.

## 3 Results

### 3.1 Search results

In accordance with the established search strategy, 2,325 publications were identified through database searches. After removing duplicate entries, an initial screening was conducted by reviewing the titles and abstracts (Figure 1). Subsequently, 54 articles were selected for full-text examination, and ultimately, 12 articles fulfilled the inclusion criteria. Among the included studies, three were randomized controlled trials, and nine were retrospective observational studies, encompassing 1,505 patients. Geographically, five studies were conducted in China, two in the United States, three in Turkey, and one each in Slovenia and Vietnam (Table 1).

**Figure 1:**
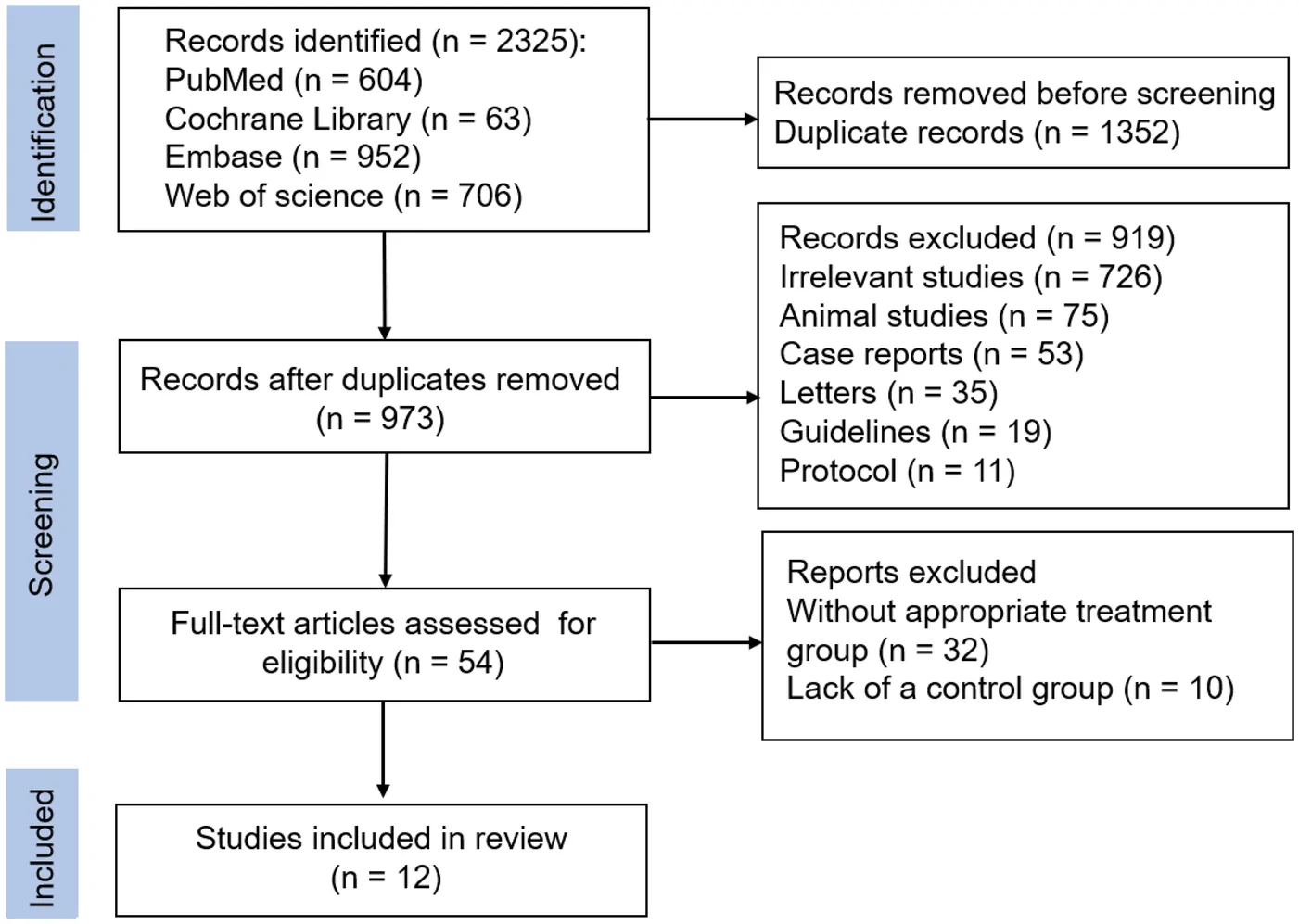
Preferred reporting items for systematic reviews and meta-analyses flow diagram.

**Table 1:** Characteristics of studies included in meta-analysis.

Study, year	Country	Study design	Therapeutic measures	Participant	Age	Triglyceride (mmol/L)	diabetes	Glucose (mmol/L)	BMI (kg/m^2^)	Drinker	Fasting protocol	Fluid resuscitation strategy
He WH 2016 (16)	China	RCT	LMWH + IIT HVHF	34 32	39.79 ± 7.84 42.34 ± 8.23	21.97 ± 8.80 22.42 ± 9.40	7 5	NA	25.20 ± 2.24 24.78 ± 2.14	10 8	NA	Conservative treatment
Jin M 2018 (17)	China	ROS	IIT + Heparin PE	34 30	34.6 ± 8.5 35.6 ± 9.9	39.4 ± 23.8 41.4 ± 37.7	18 14	NA	26.5 ± 4.4 26.6 ± 3.9	NA	NA	Fluid replacement
Frankova D 2018 (18)	America	ROS	IIT Apheresis	43 55	45.7 ± 10.8 50.3 ± 7.8	36.73 ± 16.60 38.09 ± 15.77	41 53	22.5 ± 14.8 15.9 ± 10.3		20 20	NA	NA
Yu S 2020 (19)	China	ROS	IIT PE	46 43	39.00 ± 11.01 35.72 ± 8.95	30.36 ± 11.72 29.31 ± 23.38	NA	NA	NA	NA	Restriction	Fluid resuscitation
Altinkaya E 2021 (20)	Turkey	ROS	IIT Heparin	18 18	49.94 ± 12.04 51.55 ± 11.60	30.51 ± 10.86 32.27 ± 13.98	NA	9.20 ± 2.46 12.77 ± 6.62	NA	NA	NA	NA
Araz F 2022 (21)	Turkey	ROS	IIT Apheresis	29 19	40.8 ± 7.6 39.8 ± 12.9	21.86 ± 17.54 50.67 ± 42.78	11 12	NA	NA	NA	Restriction	Fluid replacement
Gubensek J 2022 (22)	Slovenia	RCT	IIT PE	11 11	52 ± 10 50 ± 9	26 ± 8 31 ± 9	4 2	10 ± 6 8 ± 3	NA	NA	NA	Attending physician
Perez V 2023 (23)	America	ROS	IIT control	41 26	38.13 ± 9.22 40.43 ± 13.33	39.83 ± 33.10 44.72 ± 22.42	28 17	NA	34.52 ± 4.30 32.16 ± 4.16	NA	Restriction	Fluid replacement
Liu Y 2024 (24)	China	ROS	IIT control LMWH control	60 60 109 109	38.95 ± 8.59 41.15 ± 10.05 37.94 ± 8.11 39.38 ± 9.01	28.53 ± 20.71 26.48 ± 16.45 30.41 ± 22.37 22.23 ± 12.06	44 44 43 38	NA	NA	12 11 26 21	Restriction	Fluid replacement
Gulumsek, E 2024 (25)	Turkey	ROS	IIT TPE	37 18	43.53 ± 9.64 39.82 ± 3.62	40.81 ± 19.90 50.72 ± 24.21	20 8	10.66 ± 6.67 10.55 ± 7.19	27.06 ± 5.32 25.55 ± 5.06	NA	Restriction	Aggressive intravenous hydration
Thanh NH 2025 (26)	Vietnam	ROS	IIT TPE	56 33	45 ± 8.4 43 ± 9.8	48.91 ± 37.67 63.54 ± 46.50	7 10	9.71 ± 5.55 12 ± 6.97	NA	9 9	Restriction	Aggressive intravenous hydration
He W 2025 (12)	China	RCT	IIT LMWH + IIT	269 264	39.35 ± 9.69 39.35 ± 9.69	21.68 ± 7.40 20.90 ± 7.51	67 66	NA	26.35 ± 3.73 26.70 ± 2.98	86 82	Restriction	Fluid replacement

RCT, randomized controlled trial; ROS, retrospective observational study; IIT, intensified insulin therapy; HVHF, high-volume hemofiltration; LMWH, low–molecular-weight heparin; PE, plasma exchange; TPE, therapeutic plasma exchange.

The quality scores of all the literature ranged from 7 to 9 points, which was consistent with the predetermined quality criteria for this study (Table 2). Besides, the Cochrane Collaboration's tool suggested that the included RCTs were at low risk of bias (Figure 2).

**Table 2:** Methodological quality of retrospective observational studies based on the NOS.

Author Year	Newcastle-Ottawa Scale			
	Selection (maximum score 4)	Comparability (maximum score 2)	Outcome (maximum score 3)	NOS score (total score)
Jin M 2018 (17)	4	2	2	8
Frankova D 2018 (18)	4	2	2	8
Yu S 2020 (19)	4	2	3	9
Altinkaya E 2021 (20)	4	1	3	8
Araz F 2022 (21)	2	2	3	7
Perez V 2023 (23)	4	2	3	9
Liu Y 2024 (24)	4	2	3	9
Thanh NH 2025 (26)	4	2	3	9

**Figure 2:**
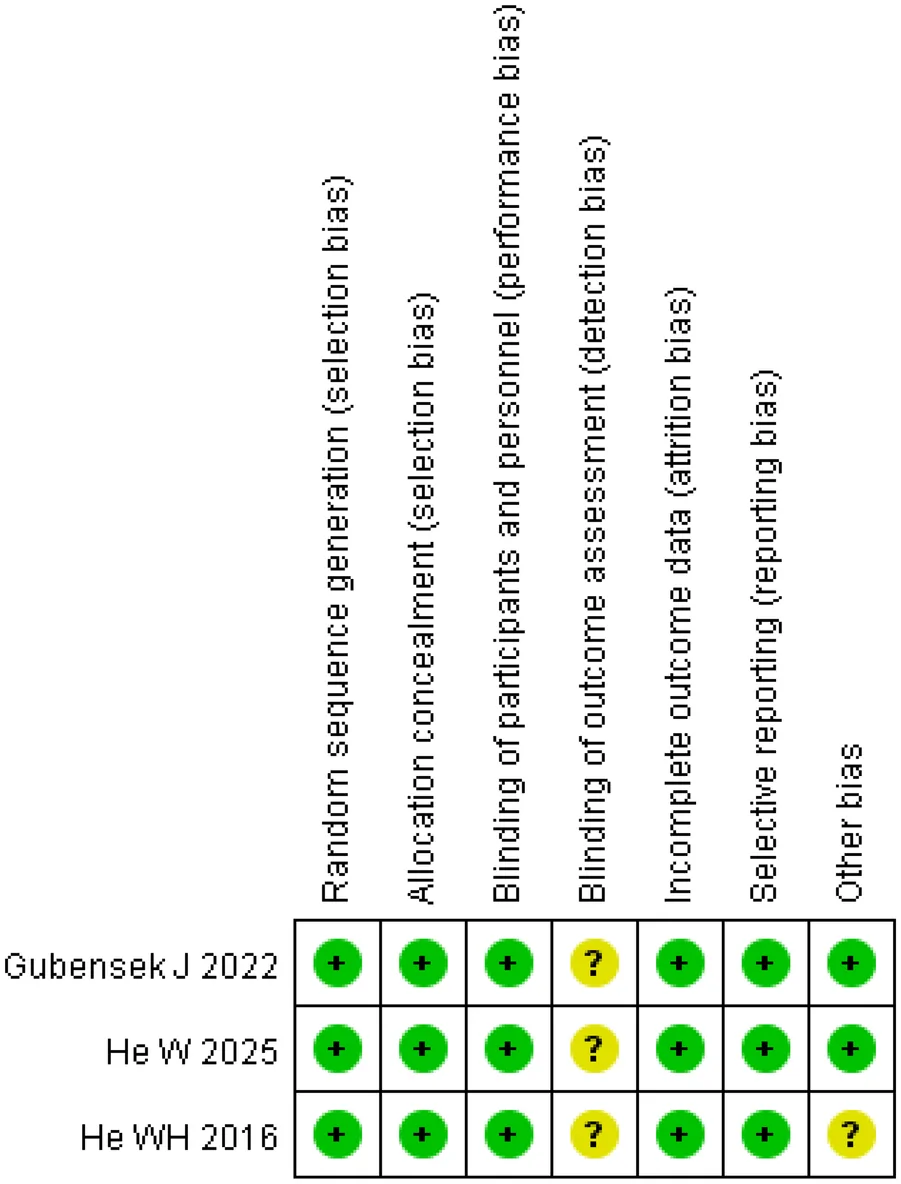
Risk of bias assessment graph of the RCTs.

These two studies, Jin M 2018 (17) and He WH 2016 (16), contribute only to the indirect comparison and not to the insulin monotherapy analyses.

### 3.2 Efficacy analysis of triglyceride levels

No statistically significant differences were observed in 24, 48, and 72 h triglyceride levels (IIT versus other therapy: 24hTG, MD: 1.25, *P* = 0.17; 48hTG, MD: 0.07, *P* = 0.88; 72hTG, MD: −0.54, *P* = 0.71) when comparing IIT with alternative treatments. Subsequent subgroup analyses revealed no significant differences in 24, 48, and 72 h triglyceride levels between IIT and blood purification (IIT versus blood purification: 24hTG, MD: 1.47, *P* = 0.29; 48hTG, MD: 0.72, *P* = 0.32; 72hTG, MD: 1.89, *P* = 0.37), nor between IIT and LMWH/heparin (IIT versus LMWH/heparin: 24hTG, MD: 1.69, *P* = 0.11; 48hTG, MD: −0.27, *P* = 0.59; 72hTG, MD: −2.62, *P* = 0.34). An adjusted indirect treatment comparison was conducted using Bucher's method (27), which calculated the indirect comparison estimates of IIT vs. IIT + LMWH/heparin by the difference between IIT and IIT + LMWH/heparin in their relative effects to the common comparator, blood purification. Indirect meta-analysis results further demonstrated no significant differences in 24 and 48 h triglyceride levels between IIT and a combination of IIT with LMWH/heparin therapy (IIT versus IIT + LMWH/heparin: 24hTG, MD: 1.36, *P* = 0.69; 48hTG, and MD: −0.45, *P* = 0.80). Additionally, the time required to achieve the target triglyceride level did not significantly differ between IIT and other treatments, including blood purification (IIT versus other therapy, SMD: −0.07, *P* = 0.69; IIT versus blood purification, SMD: 0.06, *P* = 0.87) (Figure 3). All details can be found in Supplementary Figures S1–S5.

**Figure 3:**
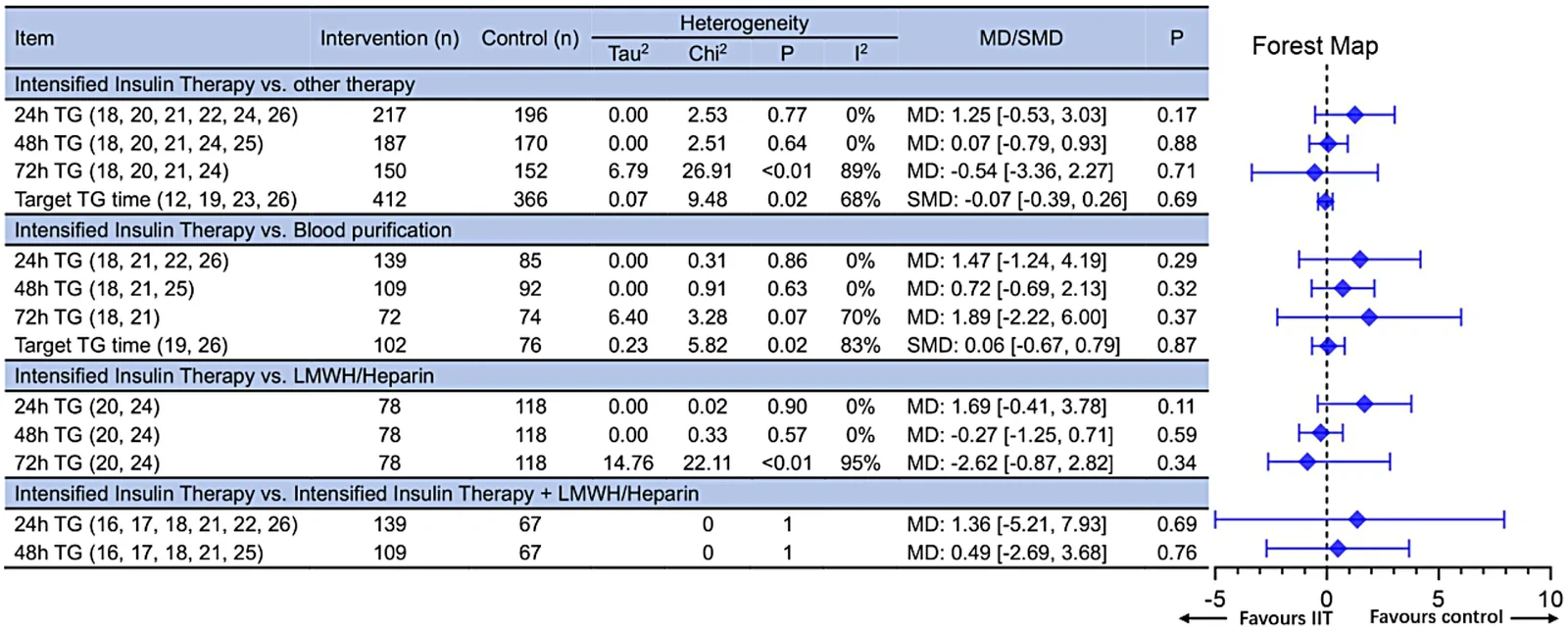
Forest plot of triglyceride levels.

### 3.3 Analysis of adverse reactions occurrence

The analysis revealed no statistically significant differences in the incidence of local pancreatic complications (RR: 0.72, *P* = 0.22), acute cystic necrosis (RR: 0.91, *P* = 0.86), and the accumulation of acute necrotic substances (RR: 0.52, *P* = 0.38) between IIT and other treatment regimens. Moreover, regarding systemic complications, there were no significant differences between IIT and alternative treatment regimens for systemic inflammatory response syndrome (RR: 1.09, *P* = 0.78), allergic reactions (RR: 0.16, *P* = 0.16), respiratory failure (RR: 0.99, *P* = 0.97), renal failure (RR: 0.52, *P* = 0.39), circulatory failure (RR: 1.24, *P* = 0.68), and mortality rates (RR: 0.52, *P* = 0.25). Furthermore, subgroup analyses indicated that IIT did not demonstrate significant differences in local pancreatic complications (RR: 0.60, *P* = 0.39), SIRS (RR: 0.80, *P* = 0.70), or mortality (RR: 0.39, *P* = 0.21) compared to the blood purification regimen (Figure 4). Details can be found in Supplementary Figures S6–S8.

**Figure 4:**
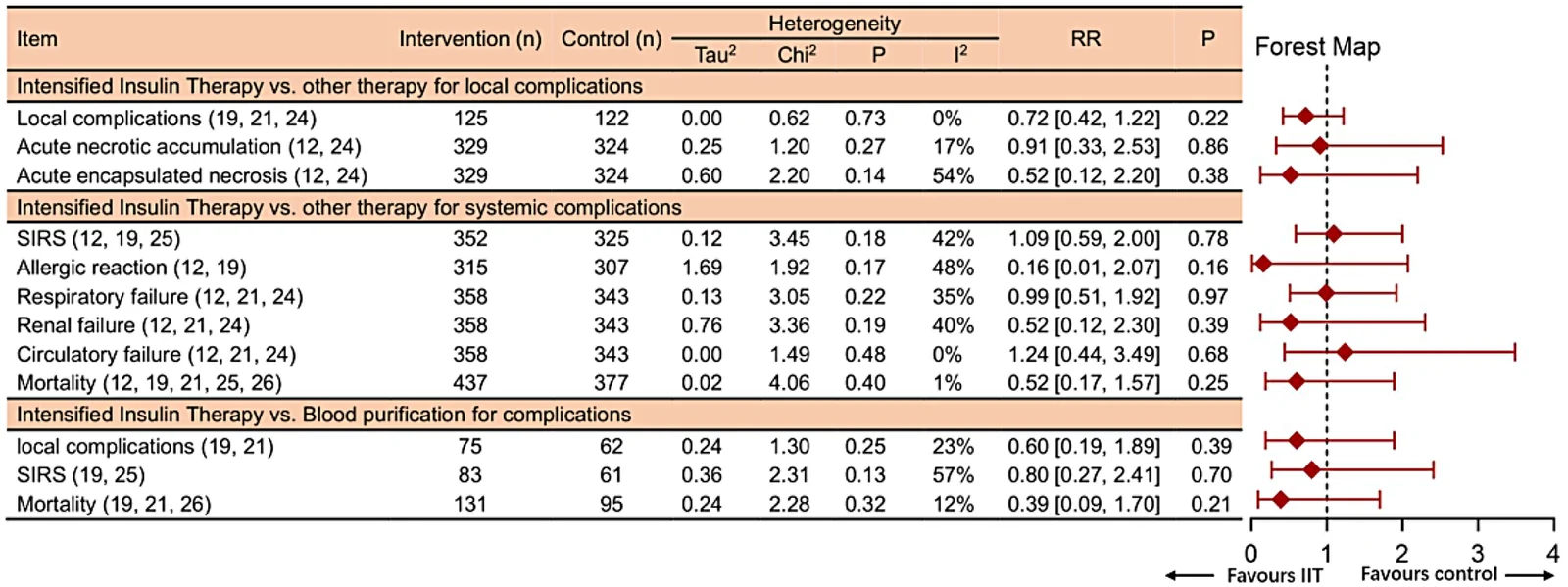
Forest plot of the incidence of adverse events.

### 3.4 Comparison of hospitalization periods

Compared with other treatment plans, intensified insulin therapy indicated no significant difference in terms of hospital stay (MD: −1.79, 95% CI: −3.78 to 0.19, *P* = 0.08). Subgroup analysis indicated that the IIT group experienced a significantly reduced duration of hospital stay in comparison to the cohort receiving the blood purification regimen (MD: −3.60, 95%CI: −5.09 to −2.11, *P* < 0.01) (Figure 5).

**Figure 5:**
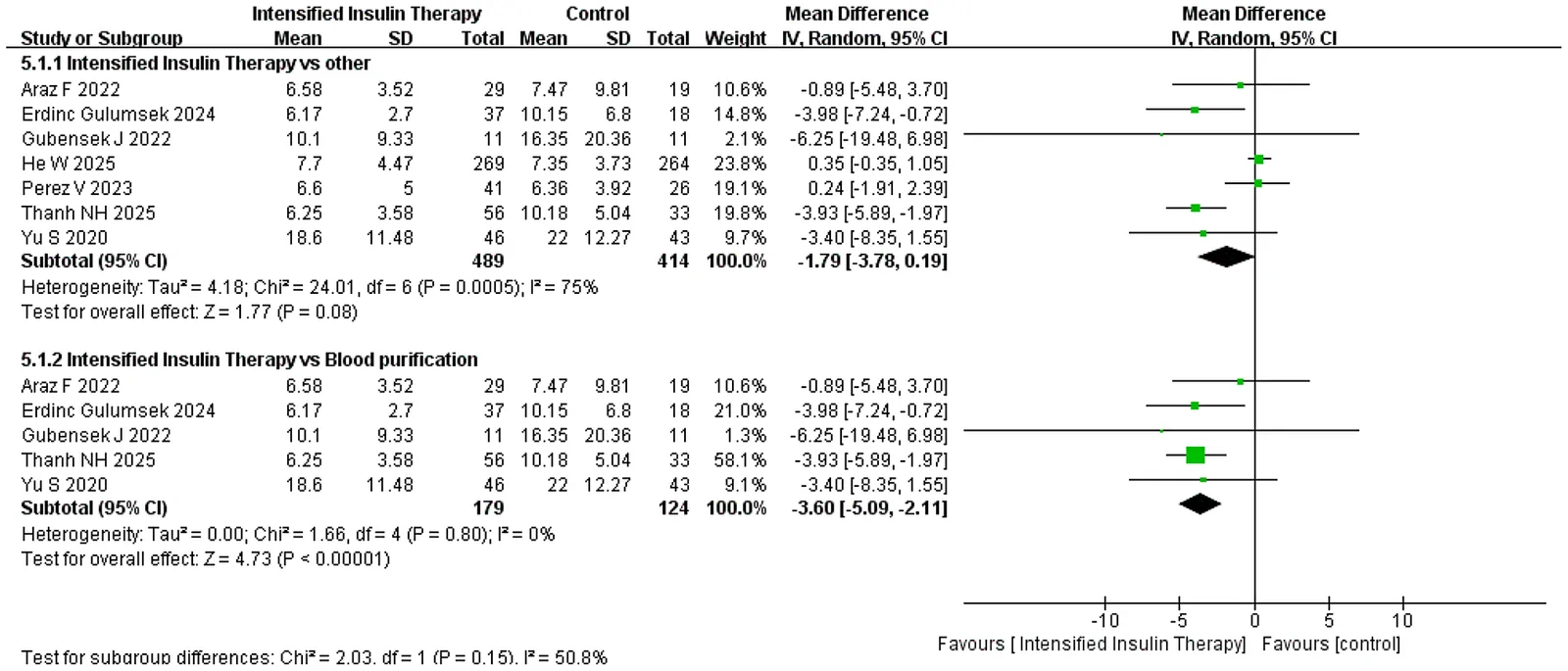
Forest plot comparing length of hospital stay.

### 3.5 Comparison of incidence of hypoglycemia

Four studies documented the incidence of hypoglycemic reactions. In one of these studies (Yu S 2020) (19), the number of events was zero in both the experimental and control groups. Consequently, risk difference (RD) was initially employed as the experimental outcome, indicating no significant difference in the incidence of hypoglycemia between the IIT group and the other treatment groups (RD: 0.00, 95%CI: −0.02 to 0.02, *P* = 0.89) (Figure 6). Excluding this study from the analysis did not yield a statistically significant difference in the incidence of hypoglycemia between the two groups (RR: 0.79, 95%CI: 0.28 to 2.25, *P* = 0.66) (Supplementary Figure S9**)**.

**Figure 6:**
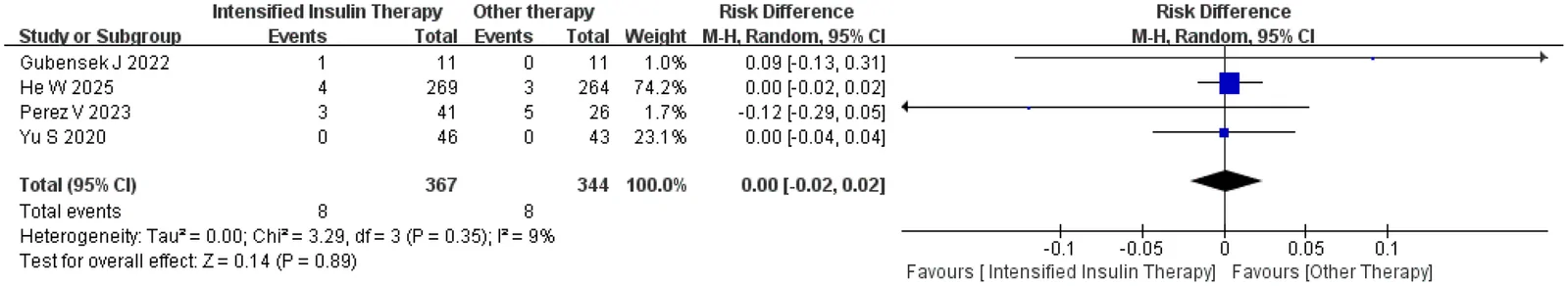
Forest plot of the incidence of hypoglycemia.

## 4 Discussion

This study encompassed 12 investigations comparing the efficacy of intensive insulin therapy with alternative treatments in patients diagnosed with HTG-AP. The findings indicated that, compared to other treatments, including blood purification therapy, intensive insulin therapy did not demonstrate a statistically significant difference in its capacity to reduce triglyceride levels. Furthermore, it was observed that intensive insulin therapy did not increase the incidence of local pancreatic complications or systemic adverse effects.

High serum triglyceride levels, usually over 11.3 mmol/L, are crucial in HTG-AP development, with increasing levels worsening the condition's severity (28, 29). Elevated plasma chylomicron concentrations contribute to increased blood viscosity, which impairs pancreatic microcirculation and induces local ischemia (30). Concurrently, excessive triglyceride degradation produces free fatty acids (FFAs), which can damage pancreatic capillaries and lead to intracellular calcium overload (31). Consequently, the rapid reduction of triglyceride levels to a safe range is currently recognized as a critical therapeutic strategy (32).

Compared with conventional treatment, blood purification can significantly reduce triglyceride levels in the short term; however, it does not improve clinical outcomes, nor does it decrease organ failure, local pancreatic complications, or the duration of hospital stay. Additionally, it is associated with higher ICU admission rates and increased hospitalization costs (33). This may be attributed to the fact that once ischemia, acidosis, and inflammatory responses are initiated within pancreatic cells, the resultant damage may persist even after the initial causes are removed, thereby leaving the overall progression of the disease unaltered (34). Consequently, the findings of the current study indicated that the duration of hospitalization was extended in the blood purification cohort compared to the IIT cohort.

Heparin, used alone or in combination with insulin, is believed to have a synergistic effect in reducing triglycerides. Nonetheless, anticoagulation therapy is associated with a modestly elevated risk of gastrointestinal and pancreatic hemorrhage in patients with AP. Additionally, while heparin can temporarily enhance triglyceride metabolism, it subsequently leads to increased hepatic degradation, resulting in reduced lipoprotein lipase activity and a rebound in triglyceride levels (9). Furthermore, recent studies indicate that in patients with HTG-AP, combining low-molecular-weight heparin (LMWH) and insulin does not significantly enhance the reduction of serum triglyceride levels compared to insulin monotherapy (12). Consequently, heparin or LMWH may not play a pivotal role in managing patients with HTG-AP.

Insulin facilitates chylomicron degradation by enhancing the lipoprotein lipase synthesis, while concurrently inhibiting lipase activity within adipocytes, thereby diminishing the release of free fatty acids (35, 36). This dual action results in a synergistic effect that contributes to the reduction of serum triglyceride levels. Besides, patients with HTG-AP frequently exhibit concomitant metabolic disorders. Previous research suggests that insulin mitigates the production of free radicals associated with these metabolic disturbances, thereby improving overall patient prognosis (37). Intensive insulin therapy demonstrates superior efficacy in lowering serum triglyceride levels. In the cohort subjected to fasting in conjunction with intensified insulin therapy, triglyceride levels were significantly reduced within the first 24 h, achieving a maximum reduction of up to 87% (38). Even in patients with extremely high levels of triglycerides, the implementation of intensive insulin therapy typically results in a reduction of triglyceride concentrations to below 5.6 mmol/L in most cases within a 72 h period (39). This rapid decrease may help prevent disease progression.

The co-occurrence of diabetic ketoacidosis (DKA) is frequently observed in patients with HTG-AP (40). Diabetic ketoacidosis can precipitate insulin deficiency, which in turn enhances lipolysis, leading to a marked elevation in triglyceride levels. Furthermore, the presence of ketoacidosis may exacerbate the severity of pancreatitis (41). In cases of DKA or hyperglycemic hyperosmolar nonketotic syndrome, intensified insulin therapy is typically employed (42). This intensified insulin therapy offers distinct advantages for affected patients.

While there is concern that intensified insulin therapy in HTG-AP may induce hypoglycemia, this risk can be effectively mitigated through diligent monitoring of blood glucose levels (43). In summary, intensified insulin therapy provides clear benefits over other treatment options in terms of cost and safety. Specifically, compared to plasma exchange, it not only achieves similar results in lowering triglyceride levels but also offers advantages such as reduced hospitalization costs and fewer adverse reactions (21).

This study has several limitations. In the context of research on HTG-AP, there is a notable deficiency in high-quality RCTs. Most existing studies are retrospective, and the overall quality of the included studies warrants improvement. Publication bias was not evaluated because the number of included studies for each outcome index was less than 10, and most of them were retrospective observational studies, with differences in baseline levels. Despite the absence of a significant difference in TG levels between the ITT and blood purification groups at 24, 48, and 72 h, the interpretation of these outcomes is constrained by the imbalance in baseline TG levels. Subsequent analysis of TG reduction at 24 h revealed that the percentage decrease in TG levels was greater in the blood purification group compared to the IIT group (Supplementary Figure S10). Furthermore, limitations inherent to secondary data analysis preclude comparison of outcomes such as bleeding, ICU admission rate and ICU length of stay, and the need for additional surgical interventions.

## 5 Conclusions

The findings of this study indicate no statistically significant difference in triglyceride reduction among intensified insulin therapy, blood purification, and the combined use of low-molecular-weight heparin. Furthermore, when comparing the insulin treatment regimen with other therapeutic approaches, no significant difference was observed in the incidence of adverse reactions. Current evidence does not demonstrate the superiority of either IIT or blood purification, but IIT may shorten the length of hospital stay to a certain extent.

## Data Availability

The original contributions presented in the study are included in the article/Supplementary Material, further inquiries can be directed to the corresponding author.
