# Supplementary material for: Evaluating the efficacy of intensified insulin therapy in hypertriglyceridemic acute pancreatitis management: a systematic review and meta-analysis
Source: Front Med (Lausanne). 2026 Sep 17;13:1944464. doi: 10.3389/fmed.2026.1944464 (PMC13626910; doi:10.3389/fmed.2026.1944464)
Supplement: Supplementary file 1 [file Supplementaryfile1.docx]

PubMed

Search Strategy: (((((insulin) AND (Hypertriglyceridemic acute pancreatitis)) OR (hypertriglyceridemia-induced acute pancreatitis)) OR (Hypertriglyceridemia-Induced Pancreatitis)) OR (Hypertriglyceridemia-Associated Acute Pancreatitis)) OR (hyperlipidemic severe acute pancreatitis)

Result: 604

Cochrane Library

Search Strategy: (((((insulin) AND (Hypertriglyceridemic acute pancreatitis)) OR (hypertriglyceridemia-induced acute pancreatitis)) OR (Hypertriglyceridemia-Induced Pancreatitis)) OR (Hypertriglyceridemia-Associated Acute Pancreatitis)) OR (hyperlipidemic severe acute pancreatitis)

Result:63

Embase databases

Search Strategy: (((((insulin) AND (Hypertriglyceridemic acute pancreatitis)) OR (hypertriglyceridemia-induced acute pancreatitis)) OR (Hypertriglyceridemia-Induced Pancreatitis)) OR (Hypertriglyceridemia-Associated Acute Pancreatitis)) OR (hyperlipidemic severe acute pancreatitis)

Result: 952

Web of Science

Search Strategy: (((((insulin) AND (Hypertriglyceridemic acute pancreatitis)) OR (hypertriglyceridemia-induced acute pancreatitis)) OR (Hypertriglyceridemia-Induced Pancreatitis)) OR (Hypertriglyceridemia-Associated Acute Pancreatitis)) OR (hyperlipidemic severe acute pancreatitis)

Result: 706
